# Multiscale Cross-Approximate Entropy Analysis as a Measurement of Complexity between ECG R-R Interval and PPG Pulse Amplitude Series among the Normal and Diabetic Subjects

**DOI:** 10.1155/2013/231762

**Published:** 2013-09-23

**Authors:** Hsien-Tsai Wu, Chih-Yuan Lee, Cyuan-Cin Liu, An-Bang Liu

**Affiliations:** ^1^Department of Electrical Engineering, National Dong Hwa University, Hualien 97401, Taiwan; ^2^Department of Neurology, Buddhist Tzu Chi General Hospital and Buddhist Tzu Chi University, No. 707, Section 3, Chung-Yang Road, Hualien 97074, Taiwan

## Abstract

Physiological signals often show complex fluctuation (CF) under the dual influence of temporal and spatial scales, and CF can be used to assess the health of physiologic systems in the human body. This study applied multiscale cross-approximate entropy (MC-ApEn) to quantify the complex fluctuation between R-R intervals series and photoplethysmography amplitude series. All subjects were then divided into the following two groups: healthy upper middle-aged subjects (Group 1, age range: 41–80 years, *n* = 27) and upper middle-aged subjects with type 2 diabetes (Group 2, age range: 41–80 years, *n* = 24). There are significant differences of heart rate variability, LHR, between Groups 1 and 2 (1.94 ± 1.21 versus 1.32 ± 1.00, *P* = 0.031). Results demonstrated differences in sum of large scale MC-ApEn (MC-ApEn_LS_) (5.32 ± 0.50 versus 4.74 ± 0.78, *P* = 0.003). This parameter has a good agreement with pulse-pulse interval and pulse amplitude ratio (PAR), a simplified assessment for baroreflex activity. In conclusion, this study employed the MC-ApEn method, integrating multiple temporal and spatial scales, to quantify the complex interaction between the two physical signals. The MC-ApEn_LS_ parameter could accurately reflect disease process in diabetics and might be another way for assessing the autonomic nerve function.

## 1. Introduction

Under the influences of temporal and spatial scales, physiological signals often show complex fluctuation (CF) [1, 2]. The reduced CF is frequently found in the aged or diseased. This finding indicates that decreased adaptability of physiologic systems is an aging or pathological phenomenon [1]. There are several traditional entropy-based assessments, such as approximate entropy (ApEn), sample entropy (SampEn), Shannon entropy, and Kolmogorov-Sinai (KS) entropy, used to quantify the complexity of a single physiological signal [3]. Koskinen et al. [4] used ApEn to analyze the electroencephalographic (EEG) signals of anesthetized subjects. The results showed that EEG signals were more complex when the subject were in a conscious state than when they were in an unconscious state. Alcaraz and Rieta [5] used SampEn to analyse the electrocardiographic (ECG) recordings of patients with atrial fibrillation (AF). The results demonstrated a gradual decrease in the CF of ECG signals 60 minutes prior to the onset of AF. However, homeostasis of an organism is the dynamic balance of multiple physiological systems. Simultaneous assessment of complex multimodal signals is approaching the real physiological phenomena and may offer a more sensitive detection for aging or pathological processes. For example, cardiopulmonary coupling, by measuring the interaction between ECG R-R interval (RRI) and respiratory time series, has decreased in the untreated patients with major depression as compared with that in the treated subjects [6]. Cross-approximate entropy (C-ApEn) [6–11] can be used more effectively to analyze the complex interaction between two simultaneous physiological signals [12].

With regard to multiple temporal scales, physiological signals are affected differently by the environment at different points in time [1]. Analysis of the complexity of physiologic systems should not be limited to a single scale, because results under different temporal scales may provide varying but equally valuable physiological data. Multiple temporal scales should be applied when analysing the complexity of physiological signals [13]. Previous studies have proposed methods of integrating multiscale analysis into the multiscale entropy (MSE) of SampEn to evaluate the CF of physiological signals under various temporal scales [14–17].

We have used a multiscale cross-approximate entropy (MC-ApEn) method to assess two physiological signals, RRI and pulse transit time, simultaneously, and examine the effects of multiple temporal and spatial scales. It clearly demonstrates a reduction of complexity of these two signals among the aged and diabetic [13]. In this study, we used MC-ApEn to quantify the complex interaction between other physiological signals (RRI series and photoplethysmography amplitude series, PPGA), in order to differentiate physical well-being between upper middle-aged diabetes and age-matched controls. 

## 2. Methods

### 2.1. Subject Populations and Experiment Procedure

Between July 2009 and March 2012, a total of 51 volunteers were recruited for this study. All diabetic subjects were enrolled from the Hualien Hospital Diabetic Outpatient Clinic; healthy controls were recruited from a health examination program at the same hospital. All subjects were then divided into the following two groups: healthy upper middle-aged subjects (Group 1, age range: 41–80 years, *n* = 27) and upper middle-aged subjects with type 2 diabetes (Group 2, age range: 41–80 years, *n* = 24). None of the healthy subjects had personal or family history of cardiovascular diseases. Type 2 diabetes was diagnosed as either fasting sugar higher than 126 mg/dL or HbA1c≧6.5%. All diabetic subjects had been receiving regular treatment and follow-up care in the clinic for more than two years. This study was approved by the Institutional Review Board of Hualien Hospital and National Dong Hwa University. All subjects refrained from caffeinated beverages and theophylline-containing medications for 8 hours prior to the hospital visit. Each subject gave informed consent, completed questionnaires on demographic data and medical history, and underwent blood sampling prior to data acquisition. The blood tests were administered to each subject including glycosylated hemoglobin (HbA1c), fasting blood sugar, high-density lipoprotein (HDL), low-density lipoprotein (LDL), triglyceride, and cholesterol. All subjects were permitted to rest in a supine position in a quiet, temperature-controlled room at 25 ± 1°C for 5 minutes prior to subsequent 30-minute measurements. Blood pressure was obtained once from the left arm of supine subjects using an automated oscillometric device (BP3AG1, Microlife, Taiwan) with a cuff of appropriate size, followed by the acquisition of waveform data from the index finger using a six-channel electrocardiogram-based pulse wave velocity measurement system as previously described [18, 19]. 

### 2.2. Data Acquisition

Digital volume pulse signals of PPG were acquired by an infrared sensor and attached to left index finger. The pulse signals were transmitted to two-order band-pass filter at frequency of 0.48 to 10 Hz and a low-pass filter at frequencies of 10 Hz. The ECG signals were acquired in lead II and transmitted to a notch filter set at 59 to 61 Hz and a band-pass filter at frequencies of 0.98 to 19.4 Hz. In order to store and analyze the PPG and ECG signals, a USB-6009 DAQ (National Instruments, Austin, TX, USA) was used for converting these two signals to digital signals and transmitting them to a personal computer at frequency of 500 Hz. In the end, we used LabVIEW 8.6 software (National Instruments, Austin, TX, USA) to monitor the ECG and PPG signals simultaneously.

### 2.3. The Measurement of RRI and PPGA Series

**Figure 1:**
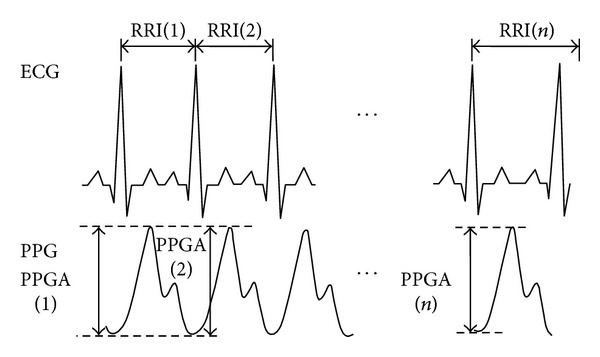
1000 consecutive data points from ECG signals and PPG signals.

For the PPG signals, the potential difference between the peak and the valley, which was prior to the peak, was defined as the pulse amplitude of PPG signals. The time difference between the two continous peaks of ECG R wave was defined as RRI(*i*), and the amplitude difference of each PPG pulse wave was defined as PPGA(*j*), as shown in Figure 1. The data length of the series in this study was set at *n* = 1000.

### 2.4. Data Detrending and Normalization

Due to a trend within physiological signals [1, 20], nonzero means may be included; therefore, we used empirical mode decomposition (EMD) [21] to deconstruct the RRI(*i*) and PPGA(*j*) series, thereby eliminating the trend from the original series. We then normalized the RRI(*i*) and PPGA(*j*) series for 1000 consecutive data points, as shown in the following:

(1) $$\begin{array}{c} \text{nRRI}\left(i\right)=\frac{\text{RRI}\left(i\right)-\bar{\text{RRI}}}{\text{S}{\text{D}}_{\text{RRI}}},\\ \text{nPPGA}\left(j\right)=\frac{\text{PPGA}\left(j\right)-\bar{\text{PPGA}}}{\text{S}{\text{D}}_{\text{PPGA}}}. \end{array}$$

In these equations, SD_RRI_ and SD_PPGA_ represent the standard deviations of 1000 data points of RRI(*i*) and PPGA(*j*), respectively. Also, $\bar{\text{RRI}}$ and $\bar{\text{PPGA}}$ represent the mean of 1000 data points of series RRI and PPGA, respectively. Complexity analysis was performed on the normalized results, nRRI(*i*) and nPPGA(*j*). The *i* and *j* represent the *i*th point of the nRRI series and the *j*th point of the nPPGA series, respectively. 

### 2.5. Multiple Spatial Scale Analysis Used in C-ApEn

Previous studies [10, 22] have used C-ApEn, an improved analysis method of approximate entropy, to analyze two synchronous physiological time series, define their relationship, and calculate the complexity within that relationship [23, 24]. This method utilizes the dynamic changes between the two series to evaluate the physiological system. Similarities between changes in the two series can be used to observe the regulatory mechanisms in the physiological system. To obtain a deeper understanding of the complexity of the physiological system, we utilized nRRI and nPPGA series to calculate the C-ApEn, using (6). The details of the whole algorithm are as follows [25].

**Step 1**

For given *m*, for two sets of *m*-vectors,

(2) x(i)≡[nRRI(i)nRRI(i+1)⋯nRRI(i+m−1)],               1≤i≤N−m+1, i∈N,y(j)≡[nPPGA(j)nPPGA(j+1)⋯nPPGA(j+m−1)],                  1≤j≤N−m+1, j∈N.

**Step 2**

Define the distance between the vectors **x**(*i*) and **y**(*j*) as the maximum absolute difference between their corresponding elements as follows:

(3) d[x(i),y(j)]  = max⁡k=1m[|nRRI(i+k−1)−nPPGA(j+k−1)|].

**Step 3**

With the given **x**(*i*), find the value of *d*[**x**(*i*), **y**(*j*)] (where *j* = 1 to *N* − *m* + 1) that is smaller than or equal to *r* and the ratio of this number to the total number of *m*-vectors (*N* − *m* + 1). That is, let *N*_nRRI  nPPGA_^*m*^(*i*) equal the number of **y**(*j*) satisfying the requirement *d*[**x**(*i*), **y**(*j*)]≦*r*; then

(4) $$\begin{array}{c} C_{\text{nRRI}\;\;\text{nPPGA}}^{m}\left(i\right)=\frac{N_{\text{nRRI}\;\;\text{nPPGA}}^{m}\left(i\right)}{N-m+1}. \end{array}$$

*C*_nRRI  nPPGA_^*m*^(*i*) measures the frequency of the *m*-point nPPGA pattern being similar (within a tolerance of ±*r*) to the *m*-point nRRI pattern formed by **x**(*i*).

**Step 4**

Average the logarithm of *C*_nRRI  nPPGA_^*m*^(*i*) over *i* to obtain *ϕ*_nRRI  nPPGA_^*m*^(*r*) as follows:

(5) $$\begin{array}{c} \phi_{\text{nRRI}\;\;\text{nPPGA}}^{m}\left(r\right)=\frac{1}{N-m+1}\sum_{i=1}^{N-m+1}\ln C_{\text{nRRI}\;\;\text{nPPGA}}^{m}\left(i\right). \end{array}$$

**Step 5**

Increase *m* by 1 and repeat Steps 1–4 to obtain *C*_nRRI  nPPGA_^*m*+1^(*i*), and *ϕ*_nRRI  nPPGA_^*m*+1^(*r*).

**Step 6**

Finally, take C-ApEn_nRRI  nPPGA_(*m*, *r*) = lim⁡_*N*→*∞*_[*ϕ*_nRRI  nPPGA_^*m*^(*r*) − *ϕ*_nRRI  nPPGA_^*m*+1^(*r*)] for ideal case. For *N*-point data, the estimate is

(6) C-ApEnnRRI nPPGA(N,m,r)  =ϕnRRI nPPGAm(r)−ϕnRRI nPPGAm+1(r),

where *m* represents the chosen vector dimension, *r* represents a tolerance range, and *N* is the data length. From Pincus's publication [26] to effectively distinguish two data series by cross-approximate entropy, it would be better to set *N*≧1000,  *m*≧2, and *r*≧0.1. To ensure efficiency and accuracy of calculation, the parameters of this study were set at *N* = 1000, *m* = 2, and *r* = 0.15.

### 2.6. Multiple Temporal Scale Analysis Used in MC-ApEn

Multiple analysis involves the use of a scale factor *τ* (*τ* = 1,2, 3,…, *n*), which is selected according to a 1-D series of consecutive cycles. This factor enables the application of a coarse-graining process capable of deriving a new series prior to the calculation of entropy in each new individual series [14]. Using this approach, we performed coarse-graining on the normalized 1-D consecutive cycles of the nRRI(*i*) and nPPGA(*j*) series based on scale factor *τ*, thereby obtaining the series nRRI(*i*) and nPPGA(*j*) as shown in (7). We then calculated as follows:

(7) nRRI(u)(τ)=1τ∑i=(u−1)τ+1uτnRRI(i), 1≤u≤1000τ, u∈N,nPPGA(u)(τ)  =1τ∑j=(u−1)τ+1uτnPPGA(j), 1≤u≤1000τ, u∈N.

Repeat Steps 1–6 to calculate MC-ApEn index in scales 2–6. The values of C-ApEn_nRRI  nPPGA_(*τ*) were obtained from a range of scale factors between 1 and 6 using the MC-ApEn data analysis method. The summation values of C-ApEn_nRRI  nPPGA_(*τ*) between scale factors 1 and 3 were defined as small scale; those between scale factors 4 and 6 were defined as large scale [27]. The sum of C-ApEn between scale factors 1 and 3 was defined as MC-ApEn_SS_ in (8), while the sum of C-ApEn between scale factors 4 and 6 was defined as MC-ApEn_LS_ in (9). Defining and calculating these two indices of multiscale cross-approximate entropy enable the assessment and quantification of complexity in RRI and PPGA between different scale factors as follows:

(8) $$\begin{array}{c} \text{MC-ApE}{\text{n}}_{\text{SS}}=\sum_{\tau =1}^{3}\text{C-ApE}{\text{n}}_{\text{nRRI}\;\;\text{nPPGA}}\left(\tau \right), \end{array}$$

(9) $$\begin{array}{c} \text{MC-ApE}{\text{n}}_{\text{LS}}=\sum_{\tau =4}^{6}\text{C-ApE}{\text{n}}_{\text{nRRI}\;\;\text{nPPGA}}\left(\tau \right). \end{array}$$

### 2.7. MSE of RRI and PPGA Series

To assess the complexity of RRI and PPGA series, sample entropy was used for multiscale analysis in six scales [16]. The results of sample entropy between scale factors 1 and 3 were defined as small scale (SS), and those between scale factors 4 and 6 were defined as large scale (LS). The sum of MSE in small scale of RRI and PPGA series was defined as MSE_RRI, SS_ and MSE_PPGA, SS_, respectively. Similarly, the sum of MSE in large scale of RRI and PPGA series was defined as MSE_RRI, LS_ and MSE_PPGA, LS_, respectively. 

### 2.8. Analysis of Conventional Heart Rate Variability

Through applying frequency domain analysis of R-R interval series by fast Fourier transform, heart rate variability (HRV) was used for assessing autonomic function in this study. A low-frequency power (LFP) was defined as the total power between the frequencies at range of 0.04 to 0.15 Hz; also a high frequency power (HFP) was defined as the total power between the frequencies at range of 0.15 to 0.4 Hz. Furthermore, the ratio of LFP to HFP was defined as LHR (the LFP/HFP ratio), a useful indicator of cardiac autonomic function.

### 2.9. Pulse-Pulse Interval and Amplitude Ratio (PAR)

Using half of the maximal value during the measurement as the low threshold, we applied the first derivative equal to zero as the local maximum of each PPG pulse signal, which we regarded as the peak of each PPG pulse wave. Then, PPI was defined as the time interval between two adjacent peaks of the PPG signals. PAR was measured by spontaneous sequence technique as our previous publication [28]. The correlation coefficient of nRRI(*i*), and nPPGA(*j*) was defined as *R*. We derived *R* value for the number of sets of the three consecutive increasing nRRI(*i*) and nPPGA(*j*), and calculated the slope of each set through the whole data points. The PAR was calculated as the mean of all the slopes, while *R* value was bigger than 0.9. 

### 2.10. Statistical Analysis

Average values were expressed as mean ± SD. Significant differences in anthropometric, hemodynamic, and computational parameters (i.e., MSE(RRI), MSE(PPGA), MC-ApEn_LS_, and MC-ApEn_SS_) between different groups were determined using an independent sample *t*-test, when the analysis data were normally distributed, and if the analysis data were not normally distributed, we used the nonparametric Mann-Whitney *U* test. To assess the agreement of the MC-ApEn and the PAR, we adopted the Bland-Altman method [29] to measure agreement between the two parameters. Statistical package for the social science (SPSS, version 14.0 for Windows) was used for all statistical analysis. A *P* value less than 0.05 was considered statistically significant. 

## 3. Results

### 3.1. Demographic Data and Blood Biochemical Parameters between the Two Groups

To control the effect of age, we recruited healthy subjects at upper-middle age (Group 1) and age-matched diabetics (Group 2).  Table 1 presents significant differences in physical parameters, such as waist circumference (85.89 ± 10.40 versus 94.17 ± 12.27, *P* = 0.012) and pulse pressure (42.93 ± 10.37 versus 50.88 ± 13.53, *P* = 0.022), and biochemical parameters including HbA1c (5.88 ± 0.33 versus 9.09 ± 1.84, *P* < 0.001) and fasting blood sugar (99.07 ± 15.85 versus 167.21 ± 56.67, *P* < 0.001), between the two groups.

### 3.2. Result of Multiscale Cross-Approximate Entropy Analysis for RRI and PPGA Series in Six Scales

**Figure 2:**
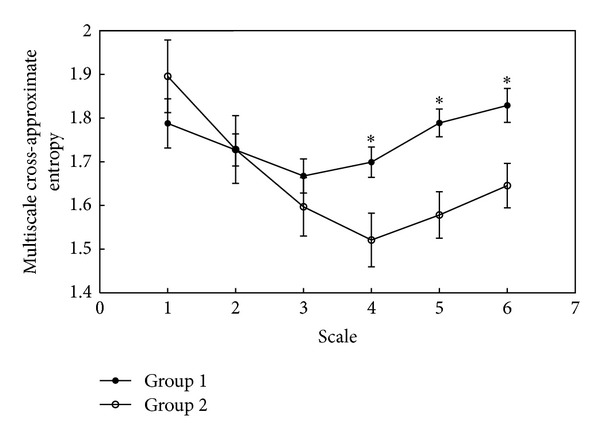
Result of multiscale cross-approximate entropy analysis for RRI and PPGA series in six scales.

The result of multiscale cross-approximate entropy analysis by using RRI and PPGA series, shown in Figure 2, represents significant differences between Groups 1 and 2 in scale factors 4 to 6. 

### 3.3. Comparisons of the Complexity of Physiological Series, PAR, and HRV between Groups 1 and 2 and Agreement between PAR and MC-ApEn_LS_

**Figure 3:**
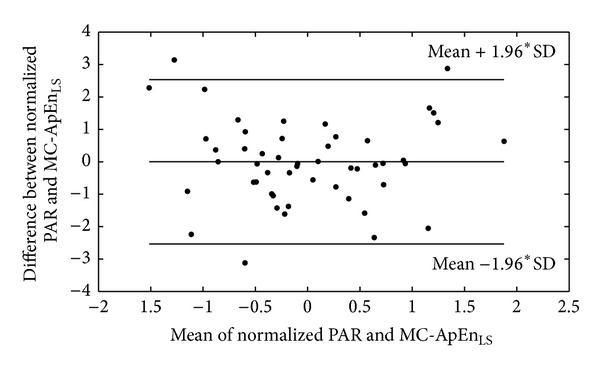
The Bland-Altman plot of normalized PAR and MC-ApEn_LS_.

In Table 2, there are significant differences in MSE_RRI, LS_ (5.28 ± 0.47 versus 4.85 ± 0.88, *P* = 0.038), MSE_PPGA, LS_ (4.65 ± 0.95 versus 3.93 ± 1.19, *P* = 0.017), and MC-ApEn_LS_ (5.32 ± 0.50 versus 4.74 ± 0.78, *P* = 0.003). Moreover, significant different PAR exists between Groups 1 and 2 (0.46 ± 0.14 versus 0.34 ± 0.10, *P* = 0.006). In addition, result of HRV analysis showed the difference in LHR (1.94 ± 1.21 versus 1.32 ± 1.00, *P* = 0.031), LFP (311.66 ± 274.85 versus 100.87 ± 95.96, *P* < 0.001), and HFP (206.50 ± 184.93 versus 126.02 ± 148.70, *P* = 0.024).  Figure 3 demonstrates a good agreement between MC-ApEn_LS_ and PAR after normalizing both parameters. 

## 4. Discussion

Table 1 demonstrates that the diabetics had larger waist circumference, higher pulse pressure, and glycosylated hemoglobin as compared with the healthy controls. We supposed that these diabetic patients should have higher risk of autonomic neuropathy [30] and arterial stiffness and lower complexity [16]. There are significant differences in all HRV parameters (LHR, LFP, and HFP) between these two groups (Table 2). It is similar to the findings about diabetic autonomic neuropathy [30]. Meanwhile, the assessments of arterial baroreflex, PAR, and MC-ApEn_LS_ are also different between the diabetic and healthy subjects. Previous studies have never shown decreased baroreflex activity in the diabetics [31]. Interestingly, multiscale entropy analysis of RRI and PPGA series shows significant differences of these two parameters at large scale but not at small scale between these two groups. It may suggest that diabetes mellitus decreases heart rate variability and oscillation of blood pressure [31]. Therefore, adaptive analysis of a single physiological signal with regard to multiple temporal scale can offer a more sensitive measurement to detect disease process than the traditional analyses do.

ECG and infrared digital pulse signals are frequently referred to as clinical applications. However, according to recent research [1, 2], the dual impacts of multiple temporal and spatial scales causing CF in physiological signals are always ignored in clinical work. In this study, we used the MC-ApEn method which considers the effect of multiple temporal and spatial scales when evaluating complex interaction between the RRI series and the PPGA series. Arterial baroreflex plays a key role in the homeostasis of blood pressure. It provides a negative feedback loop from the baroreceptors in the aortic arch and carotid sinuses to the brainstem. Elevated blood pressure stimulates the baroreceptors to increase parasympathetic activity and then slows the heart rate [32, 33]. Based on this physiological phenomenon, baroreflex sensitivity (BRS) has been quantified as the relationship between the increment of systolic blood pressure (SBP) and the change of interbeat intervals of the heart, which could indicate autonomic innervation of the heart. Previous study [34] showed a time lag of about 5 beats between increasing blood pressure and prolongation of RRI. In the result of MC-ApEn analysis, there is also a great difference between Groups 1 and 2 in the scales 4–6 (Figure 2). 

Recently, we proposed a simplified method to quantify the relationship between amplitude of pulse wave and pulse-pulse-interval by spontaneous sequence technique, namely, PAR. The new parameter can be used to detect early cardiac autonomic neuropathy of the diabetic subjects [28]. In fact, the relation between RRI and PPGA series in MC-ApEn_LS_ might be similar to the relation between PPI and pulse amplitude, PAR, and also the relationship between PPI and oscillation measured by conventional instruments such as Finapres [35, 36]. Through Bland-Altman analysis, we found a good agreement between PAR and MC-ApEn_LS_ (Figure 3). So, perhaps MC-ApEn_LS_ would be an effective parameter to evaluate baroreflex activity. 

 The current study suffers from a limitation. A lengthy process of data acquisition and considerable calculation and off-line processing are needed for MC-ApEn analysis as opposed to the relatively shorter duration measurement of BRS by conventional method or by our previously proposed PAR. However, MC-ApEn offers another measurement of dual interaction of blood pressure and R-R intervals in a longer period, which would be more consistent than the other two measurements do. Further pharmacological tests or long-term clinical cohort studies may provide more information for future clinical applications. 

## 5. Conclusion

In conclusion, this study employed the MC-ApEn method, which integrates multiple temporal and spatial scales, to quantify the complex interaction between RRI and PPGA series. This new parameter has a good agreement with a simplified measurement of baroreflex activities, PAR. According to our results, MC-ApEn could be used as a useful method for assessing autonomic nerve function.

## Figures and Tables

**Table 1:** Comparisons of demographic, anthropometric, and serum biochemical parameters between Group 1 and Group 2.

Parameters	Group 1 (*n* = 27) (male: 12, female: 15)	Group 2 (*n* = 24) (male: 13, female: 11)	*P* value
Age, year	54.96 ± 8.75	57.71 ± 7.50	*P* = 0.079
BMI, kg/m^2^	25.73 ± 3.71	27.56 ± 5.09	*P* = 0.145
WC, cm	85.89 ± 10.40	94.17 ± 12.27	***P*** = **0.012**
SBP, mmHg	119.52 ± 14.70	126.71 ± 16.62	*P* = 0.108
DBP, mmHg	76.59 ± 10.18	75.83 ± 9.51	*P* = 0.785
PP, mmHg	42.93 ± 10.37	50.88 ± 13.53	***P*** = **0.022**
HbA1c, %	5.88 ± 0.33	9.09 ± 1.84	***P*** < **0.001**
FBS, mg/dL	99.07 ± 15.85	167.21 ± 56.67	***P*** < **0.001**
LDL, mg/dL	124.22 ± 28.90	121.33 ± 31.55	*P* = 0.308
HDL, mg/dL	50.48 ± 19.84	42.83 ± 15.78	*P* = 0.122
Cholesterol, mg/dL	208.81 ± 36.36	199.75 ± 47.95	*P* = 0.177
Triglyceride, mg/dL	126.11 ± 97.72	164.96 ± 107.41	*P* = 0.113

Group 1: healthy upper middle-aged subjects. Group 2: upper middle-aged subjects with type 2 diabetes. BMI: body mass index. SBP: systolic blood pressure. DBP: diastolic blood pressure. PP: pulse pressure. HbA1c: glycosylated hemoglobin. FBS: fasting blood sugar. LDL: low-density lipoprotein. HDL: high-density lipoprotein. WC: waist circumference.

**Table 2:** Comparison of MC-ApEn, MSE, PAR, and HRV between Groups 1 and 2.

Parameter	Group 1	Group 2	*P *value
MC-ApEn_SS_	5.18 ± 0.59	5.22 ± 1.02	*P* = 0.869
MC-ApEn_LS_	5.32 ± 0.50	4.74 ± 0.78	***P*** = **0.003**
MSE_RRI, SS_	5.17 ± 0.48	5.11 ± 1.08	*P* = 0.509
MSE_RRI, LS_	5.28 ± 0.47	4.85 ± 0.88	***P*** = **0.038**
MSE_PPGA, SS_	4.14 ± 1.09	4.03 ± 1.21	*P* = 0.664
MSE_PPGA, LS_	4.65 ± 0.95	3.93 ± 1.19	***P*** = **0.017**
PAR	0.46 ± 0.14	0.34 ± 0.10	***P*** = **0.006**
LHR	1.94 ± 1.21	1.32 ± 1.00	***P*** = **0.031**
LFP	311.66 ± 274.85	100.87 ± 95.96	***P*** < **0.001**
HFP	206.50 ± 184.93	126.02 ± 148.70	***P*** = **0.024**

Group 1: healthy upper middle-aged subjects. Group 2: upper middle-aged subjects with type 2 diabetes. MC-ApEn: multiscale cross-approximate entropy. MSE: multiscale entropy. RRI: R-R intervals. PPGA: photoplethysmography amplitude. SS: small scale (the sum of the algorithm between scale factors 1–3). LS: large scale (the sum of the algorithm between Scale factors 4–6). PAR: pulse-pulse interval and amplitude ratio. LHR: low-frequency-power/high-frequency power ratio. LFP: low-frequency power. HFP: high-frequency power.
